# High levels of interleukin-6 in patients with rheumatoid arthritis are associated with greater improvements in health-related quality of life for sarilumab compared with adalimumab

**DOI:** 10.1186/s13075-020-02344-3

**Published:** 2020-10-20

**Authors:** Vibeke Strand, Susan H. Boklage, Toshio Kimura, Florence Joly, Anita Boyapati, Jérôme Msihid

**Affiliations:** 1 Stanford University, Palo Alto, CA USA; 2 Formerly, Regeneron Pharmaceuticals, Inc., Tarrytown, NY USA; 3 Regeneron Pharmaceuticals, Inc., Tarrytown, NY USA; 4 Sanofi, 1 Avenue Pierre Brossolette, 91385 Chilly-Mazarin, France

**Keywords:** Rheumatoid arthritis, Interleukin-6, Sarilumab, Morning stiffness, Fatigue, Physical function, Pain, Biomarkers, Health-related quality of life, Adalimumab

## Abstract

**Background:**

Increased levels of cytokines, including interleukin-6 (IL-6), reflect inflammation and have been shown to be predictive of therapeutic responses, fatigue, pain, and depression in patients with rheumatoid arthritis (RA), but limited data exist on associations between IL-6 levels and health-related quality of life (HRQoL). This post hoc analysis of MONARCH phase III randomized controlled trial data evaluated the potential of baseline IL-6 levels to differentially predict HRQoL improvements with sarilumab, a fully human monoclonal antibody directed against both soluble and membrane-bound IL-6 receptor α (anti-IL-6Rα) versus adalimumab, a tumor necrosis factor α inhibitor, both approved for treatment of active RA.

**Methods:**

Baseline serum IL-6 levels in 300/369 randomized patients were categorized into low (1.6–7.1 pg/mL), medium (7.2–39.5 pg/mL), and high (39.6–692.3 pg/mL) tertiles. HRQoL was measured at baseline and week (W)24 and W52 by Short Form 36 (SF-36) physical/mental component summary (PCS/MCS) and domain scores, Functional Assessment of Chronic Illness Therapy -fatigue, and duration of morning stiffness visual analog scale (AM-stiffness VAS). Linear regression of changes from baseline in HRQoL (IL-6 tertile, treatment, region as a stratification factor, and IL-6 tertile-by-treatment interaction as fixed effects) assessed predictivity of baseline IL-6 levels, with low tertile as reference. Pairwise comparisons of improvements between treatment groups were performed by tertile; least squares mean differences and 95% CIs were calculated. Similar analyses evaluated W24 patient-level response on minimum clinically important differences (MCID).

**Results:**

At baseline, patients with high versus medium or low IL-6 levels (*n* = 100, respectively) reported worse (nominal *p* < 0.05) SF-36 MCS and role-physical, bodily pain, social functioning, role-emotional domain, and AM-stiffness VAS scores. There was a greater treatment effect with sarilumab versus adalimumab in high tertile versus low tertile groups in SF-36 PCS, physical functioning domain, and AM-stiffness VAS (nominal interaction *p* < 0.05). PCS improvements ≥MCID were higher in high (odds ratio [OR] 6.31 [2.37, 16.81]) versus low (OR 0.97 [0.43, 2.16]) tertiles with sarilumab versus adalimumab (nominal interaction *p* < 0.05). Adverse events between IL-6 tertiles were similar.

**Conclusions:**

Patients with high baseline IL-6 levels reported better improvements in PCS, physical functioning domain, and AM-stiffness scores with sarilumab versus adalimumab and safety consistent with IL-6R blockade.

**Trial registration:**

NCT02332590. Registered on 5 January 2015

## Background

The understanding of the multifunctional role of interleukin-6 (IL-6) in biologic activities has expanded in the last decade [1, 2]. Dysregulation of IL-6 has been implicated in the onset or development of several diseases, particularly inflammatory disorders such as rheumatoid arthritis (RA) [3, 4], whereby elevated levels of IL-6 in serum, synovial fluid, and various tissues have correlated with increased RA disease activity [5, 6].

The contribution of IL-6 to joint inflammation and bone erosion in RA is well established [7]; however, it has also been associated with non-articular manifestations of RA, including anemia [8], type 2 diabetes mellitus [9], and increased cardiovascular risk [10]. IL-6 levels also associate with a number of RA-related patient-reported outcomes (PRO), including fatigue and pain [11–13]. Studies of anti-IL-6R agents, such as tocilizumab [14–21] and sarilumab [22–24], in the treatment of moderate-to-severe RA have revealed the benefits of IL-6 inhibition, not only in the reduction of disease activity, but also improvement in pain and mood disorders associated with RA. The value of these clinical and PRO data notwithstanding, a formal association between IL-6 levels and overall health-related quality of life (HRQoL) in RA patients has not been investigated to date. Given that there are two approved therapeutics for RA that specifically block IL-6 signaling, a better understanding of the association between IL-6 levels and HRQoL fatigue and morning-stiffness is warranted as a potential biomarker to guide RA clinical decision-making.

Sarilumab is a fully human monoclonal antibody directed against both soluble and membrane-bound IL-6 receptor α (anti-IL-6Rα); this biologic disease-modifying antirheumatic drug (bDMARD) is approved for treatment of adult patients with moderate-to-severely active RA with inadequate responses or intolerance to one or more DMARDs [25, 26]. Sarilumab can be used in combination with methotrexate or as monotherapy when treatment with methotrexate is not appropriate. The MONARCH phase III, randomized controlled trial (RCT) of sarilumab (NCT02332590), compared the efficacy and safety of subcutaneous (SC) sarilumab 200 mg monotherapy every 2 weeks (q2w) versus adalimumab 40 mg SC monotherapy q2w in patients with RA not receiving methotrexate due to intolerance or inadequate responses. Adalimumab, a tumor necrosis factor α inhibitor (TNFi) bDMARD, is approved for the treatment of active RA and can also be used in combination or as monotherapy.

The MONARCH RCT demonstrated greater reductions in disease activity and symptoms of RA [24], with greater improvements in PROs including HRQoL [27] with sarilumab versus adalimumab. Safety profiles of both therapies were consistent with previously reported data in both therapeutic classes [28–31].

The objective of these post hoc analyses was to evaluate whether baseline levels of IL-6 are associated with improvements in PROs including HRQoL with sarilumab versus adalimumab.

## Methods

### Biomarker assessments

Serum levels of IL-6 were measured using a validated enzyme-linked immunosorbent assay in 300 of 369 randomized patients in the intent-to-treat population who provided consent with at least one serum sample drawn at baseline (i.e., the biomarker population). Patients were categorized into tertiles of baseline IL-6 levels across both treatment groups, classified as low, medium, and high, based on ranges of 1.6–7.1 pg/mL, 7.2–39.5 pg/mL, and 39.6–692.3 pg/mL, respectively.

### HRQoL endpoints

Three PRO questionnaires were administered at baseline and (W)24 and W52: Short Form 36 (SF-36), Functional Assessment of Chronic Illness Therapy (FACIT)-fatigue, and duration of morning stiffness visual analog scale (AM-stiffness VAS). SF-36, scores evaluated included physical and mental component summary (PCS, MCS) and domains: physical functioning (PF), role-physical (RP), bodily pain (BP), general health (GH), vitality (VT), social functioning (SF), role-emotional (RE), and mental health (MH). Minimum clinically important differences (MCID) for these endpoints were [32] 2.5 for PCS and MCS [33], 4.0 for FACIT [34, 35], and 10.0 mm for AM-stiffness [21].

### Statistical analyses

The Kruskal-Wallis test first evaluated if patients with high baseline IL-6 levels reported worse baseline PRO scores versus those with medium or low IL-6 levels.

The ability of IL-6 levels to predict improvements in HRQoL associated with sarilumab versus adalimumab was then tested using a linear fixed effect model of change from baseline (CFB) in PRO/HRQoL scores, with IL-6 tertile, treatment, region as stratification factor, and baseline IL-6 tertile-by-treatment interactions as fixed effects. The IL-6 tertile at baseline-by-treatment interaction term was calculated using low IL-6 tertile as a reference, i.e., it specifically evaluated whether there was a greater change in PRO/HRQoL scores in patients treated with sarilumab versus adalimumab in high or medium IL-6 tertile groups, respectively, compared with the low IL-6 tertile group. Pairwise comparisons of HRQoL scores between sarilumab versus adalimumab were performed separately for each IL-6 tertile, and least squares mean (LSM) CFB and corresponding 95% confidence intervals (CI) derived.

Patient-level responses (W24 and W52) in HRQoL between sarilumab versus adalimumab were evaluated via logistic regression of within-patient improvements ≥ MCID, with treatment, region as stratification factor, IL-6 tertile at baseline, and IL-6 tertile at baseline-by-treatment interactions, specified as fixed effects. The Mantel-Haenszel estimate (stratified by the region) of odds ratio (OR) between sarilumab and adalimumab and 95% CIs were also derived in each IL-6 tertile.

As all predictive analyses were conducted post hoc, all *p* values should be considered to be nominal.

Finally, the incidences of treatment-emergent adverse events (AEs) in each IL-6 tertile were analyzed descriptively.

Analyses were performed using SAS version 9.2 or higher (SAS Institute Inc. Cary, NC).

## Results

### Analysis population

The biomarker population included 300 patients (Table 1), with 152 and 148 patients, respectively, in the adalimumab and sarilumab group. Demographics and baseline clinical characteristics between treatment arms were similar to the overall study population [24]. Mean age (standard deviation [SD]) of patients in the adalimumab and sarilumab arms, respectively, were 50.4 (± 12.5) years and 53.3 (± 12.0) years, and 78.6% and 83.7% were female. The proportion of patients in the high IL-6 tertiles in the adalimumab and sarilumab arms were 35.5% and 31.1%, respectively, 34.9% and 31.8% in medium, and 29.6% and 37.2% in low, respectively.

**Table 1:** Demographics and baseline disease characteristics of the biomarker population by treatment arm

	Biomarker population	
Baseline parameter	Adalimumab 40 mg q2w (***n*** = 152)	Sarilumab 200 mg q2w (***n*** = 148)
Age, years, mean (± SD)	53.3 (± 12.0)	50.4 (± 12.5)
Female, *n* (%)	121 (78.6)	128 (83.7)
Caucasian, *n* (%)	135 (87.7)	141 (92.2)
Duration of RA, years, mean (± SD)	6.6 (± 8.1)	7.9 (± 8.1)
Swollen joint count, mean (± SD)	17.26 (± 10.1)	18.5 (± 10.6)
Tender joint count, mean (± SD)	26.9 (± 13.9)	28.1 (± 13.4)
IL-6, pg/mL, median [Q1–Q3]	19.79 [5.86–54.59]	14.40 [4.55–47.02]
IL-6 tertile^†^, *n* (%)		
Low	45 (29.6)	55 (37.2)
Medium	53 (34.9)	47 (31.8)
High	54 (35.5)	46 (31.1)

^†^Low (1.6–7.1 pg/mL), medium (7.2–39.5 pg/mL), high (39.6–692.3 pg/mL)

*IL-6* interleukin-6, *q2w* every 2 weeks, *RA* rheumatoid arthritis, *SD* standard deviation

### Baseline disease characteristics and HRQoL scores

Patients with high baseline IL-6 levels reported worse baseline scores on SF-36 MCS and the SF, RE, RP, and BP domains, as well as AM-stiffness, compared with medium or low IL-6 tertile groups (Table 2).

**Table 2:** Baseline disease characteristics and HRQoL of the biomarker population, by IL-6 tertile

	IL-6 tertile		
	Low	Medium	High
Adalimumab 40 mg q2w, *n* (%)	45 (30)	53 (35)	54 (35)
Sarilumab 200 mg q2w, *n* (%)	55 (37)	47 (32)	46 (31)
CRP (mg/L) mean (± SD) [range]*	5.62 (9.18)	15.24 (17.14)	41.51 (34.14)
	[0.2–48.2]	[1.0–120.0]	[2.2–202.0]
ESR (mm/h) mean (± SD) [range]*	38.99 (15.56)	44.96 (20.35)	59.02 (26.48)
	[7.0–104.0]	[14.0–130.0]	[4.0–130.0]
Positive RF (> 15 IU/mL), *n* (%)**	46 (46)	72 (74)	73 (73)
Postive ACPA (≥ 17 U/mL), *n* (%)**	53 (55)	78 (81)	87 (87)
IL-6, pg/mL, median [range]	2.4 [1.6–7.1]	16.2 [7.2–39.5]	64.7 [39.6–692.3]
Baseline HRQoL scores, mean (± SD) [range]			
SF-36 summary scores (0–100)			
PCS	31.78 (6.16)	30.96 (6.25)	30.36 (6.56)
	[16.5–46.0]	[18.4–47.5]	[18.1–52.0]
MCS*	37.49 (10.47)	38.80 (12.02)	34.98 (12.61)
	[12.8–61.6]	[11.4–67.1]	[13.1–66.8]
SF-36 domain scores (0–100)			
PF	37.02 (20.01)	35.36 (19.10)	31.74 (22.36)
	[0.0–85.0]	[0.0–90.0]	[0.0–94.4]
RP*	37.56 (18.74)	35.75 (19.69)	30.44 (19.58)
	[0.0–81.3]	[0.0–100.0]	[0.0–87.5]
BP*	31.14 (15.25)	27.83 (14.42)	24.63 (16.92)
	[0.0–84.0]	[0.0–70.0]	[0.0–74.0]
GH	33.85 (15.84)	36.75 (14.78)	35.77 (17.54)
	[0.0–77.0]	[0.0–82.0]	[0.0–82.0]
VT	34.50 (16.87)	35.63 (18.04)	31.94 (17.27)
	[0.0–75.0]	[0.0–87.5]	[0.0–68.8]
SF*	48.99 (22.49)	50.63 (25.59)	41.00 (26.77)
	[0.0–100.0]	[0.0–100.0]	[0.0–100.0]
RE*	50.59 (24.26)	51.92 (26.51)	42.67 (28.70)
	[0.0–100.0]	[0.0–100.0]	[0.0–100.0]
MH	50.20 (17.95)	51.80 (20.18)	47.68 (21.00)
	[10.0–95.0]	[5.0–100.0]	[5.0–100.0]
AM-stiffness VAS (0–100 mm)*	64.60 (19.89)	68.01 (19.70)	75.17 (20.33)
	[11.0–100.0]	[10.0–100.0]	[16.0–100.0]
FACIT-fatigue (0–52)	24.12 (9.77)	24.86 (9.80)	21.89 (9.62)
	[3.0–50.0]	[1.0–48.0]	[2.0–45.0]

*ACPA* anti-citrullinated peptide antibody, *BP* bodily pain, *CRP* C-reactive protein, *ESR* erythrocyte sedimentation rate, *FACIT* Functional Assessment of Chronic Illness Therapy, *GH* general health, *HRQoL* health-related quality of life, *IL-6* interleukin-6, *MH* mental health, *AM-stiffness* duration of morning stiffness visual analog scale, *PF* physical functioning, *q2w* every 2 weeks, *RE* role-emotional, *RF* rheumatoid factor, *RP* role-physical, *SF* social functioning, *SF-36* Short Form 36, *VAS* visual analog scale, *VT* vitality

*Kruskal-Wallis test nominal *p* < 0.05

**Chi^2^ test nominal *p* < 0.05

### C-reactive protein (CRP) and erythrocyte sedimentation rate (ESR) were lower in the low IL-6 tertile; there were fewer patients in this tertile with positive rheumatoid factor and positive anti-citrullinated peptide antibody

#### Predictivity of IL-6 tertile

**Fig. 1:**
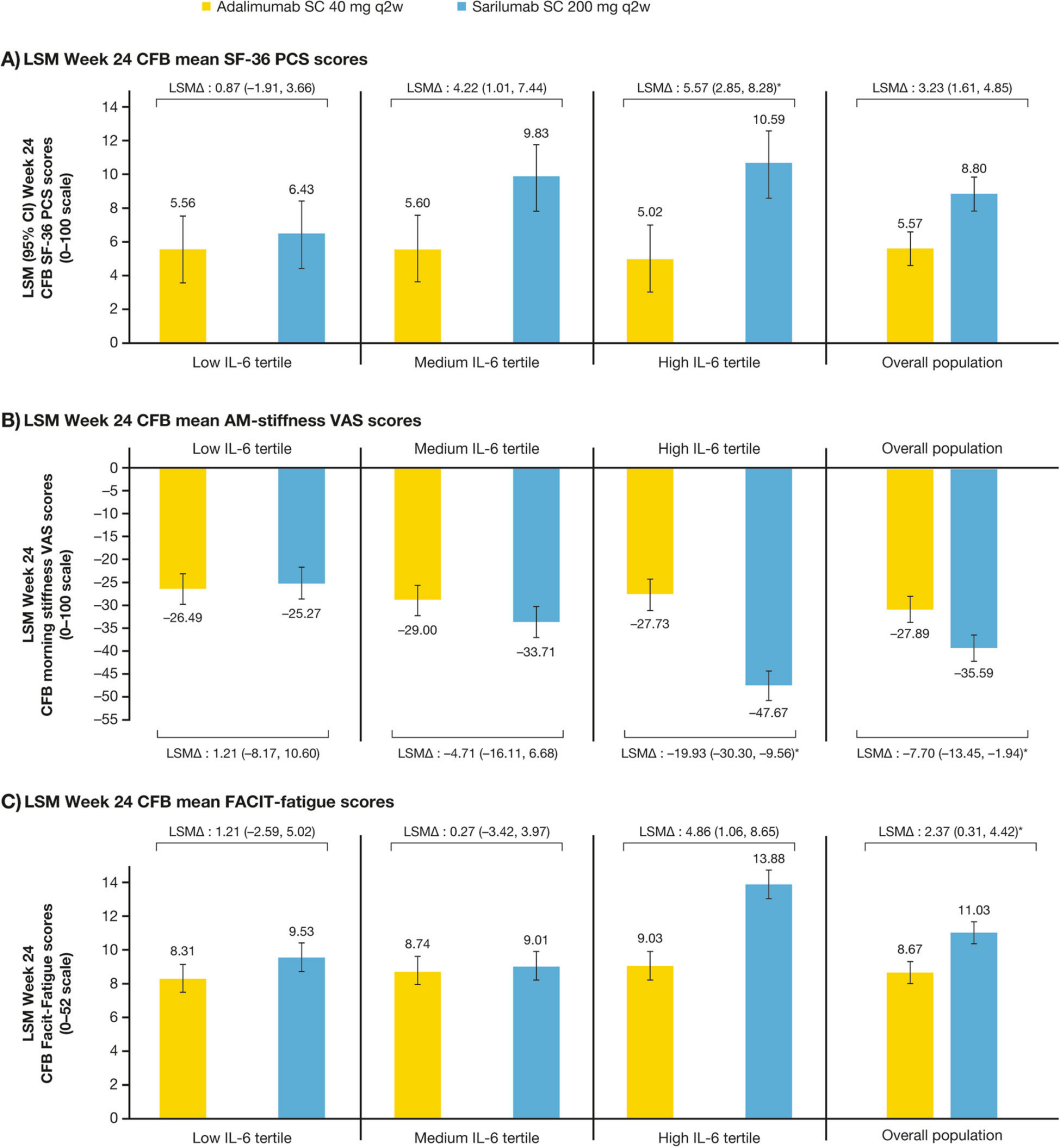
LSM change (95% CI) from baseline to week 24 on HRQoL endpoints by IL-6 tertile^†^ and overall population for SF-36 PCS scores (**a**), AM-stiffness scores (**b**), and FACIT-fatigue scores (**c**). Adalimumab: low tertile, *n* = 45; medium tertile, *n* = 53; high tertile, *n* = 54. Sarilumab: low tertile, *n* = 55; medium tertile, *n* = 47; high tertile, *n* = 46. *AM-stiffness* duration of morning stiffness visual analog scale, *CFB* change from baseline, *CI* confidence interval, *FACIT-fatigue* Functional Assessment of Chronic Illness Therapy-fatigue, *HRQo*L health-related quality of life, *IL-6* interleukin-6, *LSM* least squares mean, *LSM∆* LSM difference between sarilumab and adalimumab, *SF-36* Short Form 36, *PCS* physical component summary, *VAS* visual analog scale. ^†^Low (1.6–7.1 pg/mL), medium (7.2–39.5 pg/mL), high (39.6–692.3 pg/mL). *Nominal interaction *p* value versus low IL-6 tertile < 0.05

Nominal interaction *p* values comparing differences in HRQoL improvements in high versus low IL-6 tertiles at W24 were < 0.05 for SF-36 PCS and the PF domain, as well as for AM-stiffness. In patients with high IL-6 levels at baseline and compared with patients in the low tertile, sarilumab treatment had a larger effect on HRQoL than adalimumab, which had stable and similar effects across IL-6 tertiles. LSM differences for sarilumab versus adalimumab, respectively, in the high and low IL-6 tertiles were 5.57, 95% CI [2.85, 8.28], versus 0.87 [− 1.91, 3.66] in SF-36 PCS (Fig. 1a); 3.19 [− 4.74, 11.12] versus 16.59 [8.15, 25.03] in PF domain (data not graphed); and − 19.93 [− 30.30, − 9.56] versus 1.21 [− 8.17, 10.60] for AM-stiffness (Fig. 1b). For SF-36 MCS, interaction *p* values were ≥ 0.05, suggesting no difference in effect between high or medium IL-6 compared with low IL-6 tertile.

**Fig. 2:**
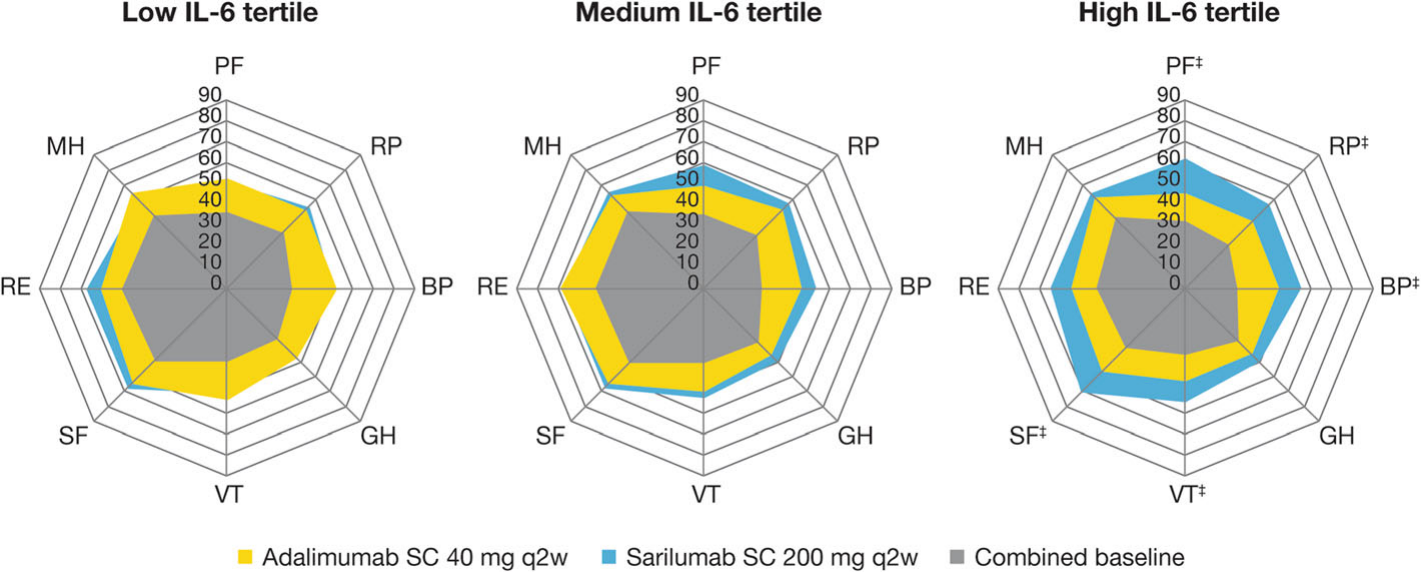
Mean SF-36 domain scores for adalimumab and sarilumab (combined baseline^†^ and week 24) by IL-6 tertile^§^. The nominal *p* value for the IL-6 tertile-by-treatment interaction using the low tertile as reference was ≥ 0.05 for all SF-36 domains except PF. ^†^Baseline combined scores are presented; change from baseline for each group cannot be inferred from the figure alone. Each 10-point interval represents twice the MCID for the SF-36 domain scores. ^‡^*p* value of the between-group difference in LSM change from baseline < 0.05 within each IL-6 tertile. ^§^Low (1.6–7.1 pg/mL), medium (7.2–39.5 pg/mL), high (39.6–692.3 pg/mL). *BP* bodily pain, *FACIT* Functional Assessment of Chronic Illness Therapy, *GH* general health, *IL-6* interleukin-6, *LSM* least squares mean, *MCID* minimal clinically important differences, *MH* mental health, *PF* physical functioning, *RE* role-emotional, *RP* role-physical, *SF* social functioning, *SF-36* Short Form 36, *VAS* visual analog scale, *VT* vitality

Regarding other SF-36 domains, all nominal interaction *p* values were < 0.05. However, there were between-group differences (nominal *p* < 0.05) for the benefit of sarilumab versus adalimumab within the high IL-6 tertile in RP, BP, VT, and SF domains, but not low or medium IL-6 tertiles (Fig. 2). Similarly, there was a difference (nominal *p* < 0.05) with sarilumab versus adalimumab within the high IL-6 tertile in FACIT-fatigue (4.86 [1.06, 8.65]), but not low or medium tertiles (Fig. 1c).

**Fig. 3:**
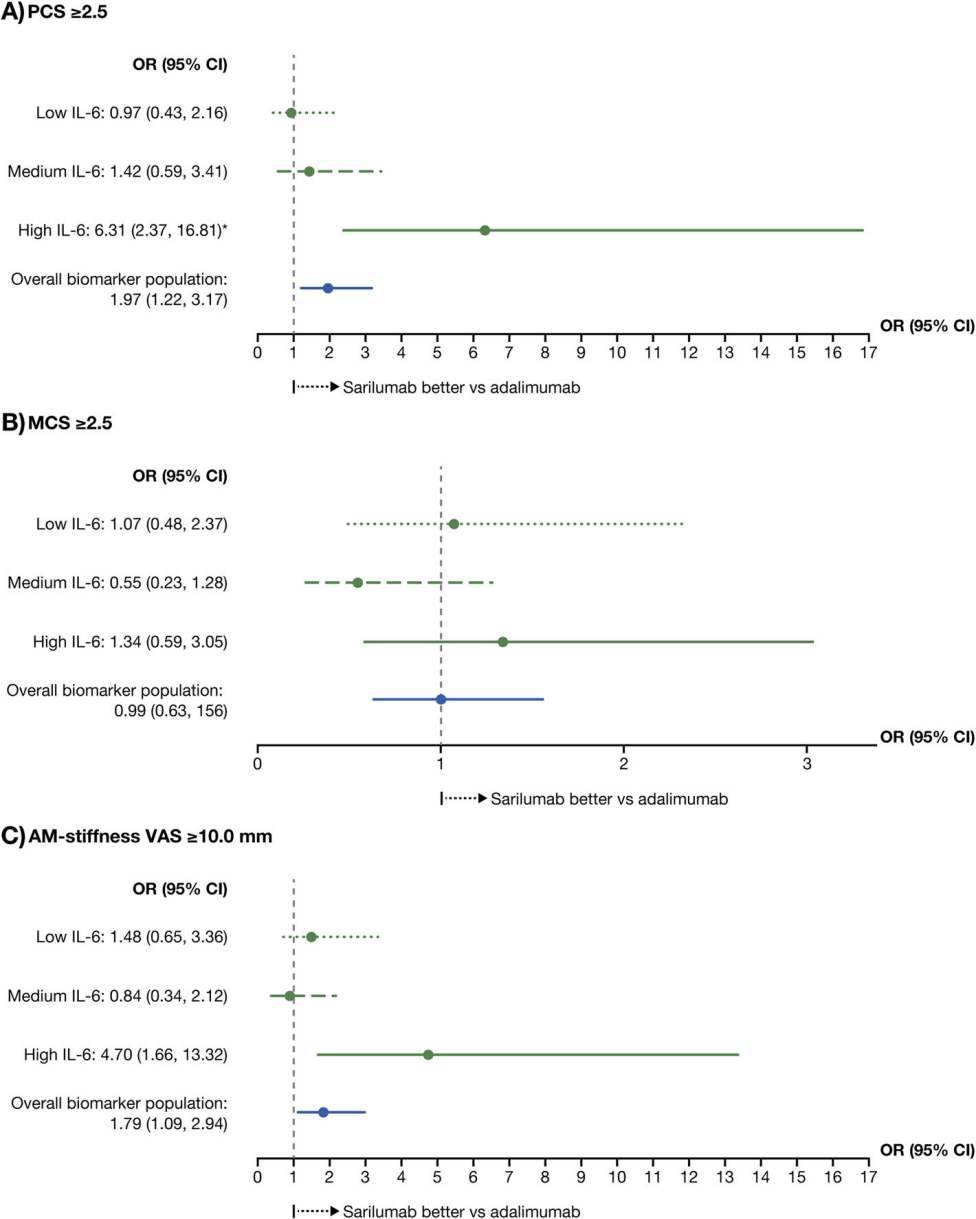
Forest plot of odd ratios from patients reporting improvements ≥ MCID by baseline IL-6 tertile for SF-36 PCS score (**a**), SF-36 MCS score (**b**), and AM-stiffness (**c**) for sarilumab 200 mg q2w versus adalimuma12321b 40 mg q2w. *Nominal *p* < 0.01 for interaction test for patients reporting improvements ≥cvbnm,./MCID (using low IL-6 tertile as the reference group). *CI* confidence interval, *MCID* minimal clinically important differences, *AM-stiffness* duration of morning stiffness, *OR* odds ratio, *PCS* physical component summary, *SF-36* Short Form 36,*VAS* visual analog scale

An IL-6 tertile at baseline-by-treatment interaction was also reported in patients reporting improvements ≥MCID in PCS scores (nominal *p* < 0.01) with high versus low IL-6 comparisons, but not other HRQoL endpoints (MCS, FACIT-fatigue, or AM-stiffness VAS). The OR and 95% CI in the high tertile was 6.31 [2.37, 16.81)] versus 0.97 [0.43, 2.16] in the low tertile (Fig. 3), indicating that patients treated with sarilumab are approximately six times more likely to report improvements in PCS scores than with adalimumab; whereas in the low tertile, there are no differences in responses.

### Safety

Descriptive analysis of AE rates indicated a similar safety profile between IL-6 tertiles [36].

## Discussion

In these analyses, at baseline, RA patients with higher levels of IL-6 reported worse PRO/HRQoL scores than medium or low levels. Differences in the treatment effect of sarilumab versus adalimumab were higher (nominal *p* < 0.05) in patients with high IL-6 versus low IL-6 levels in SF-36 PCS and PF domain scores, and AM-stiffness VAS, with a higher treatment effect in patients with elevated IL-6 values, whereas the effect of adalimumab was stable across all tertiles. Analyses of responses between IL-6 tertiles indicated that patients with high IL-6 levels were more likely to report clinically meaningful improvements in PCS scores with sarilumab versus adalimumab. While our findings suggest that IL-6 levels may be associated with those scores where larger HRQoL improvements were reported, more work is needed to better understand these impacts of disease. For example, it would be pertinent to determine an optimal cut-off for IL-6 concentration, using receiver operating characteristic (ROC) analysis for improvement in patient-level responses, or machine learning methods like Classification and Regression Trees (CART).

Although IL-6 testing is currently not standard practice, the utility of high IL-6 levels as predictive biomarkers to tailor individual therapy has been proposed to address a key goal: to determine patient-specific profiles as a means to help predict responsiveness to specific treatments [37]. To date, several biomarkers have already been associated with RA diagnosis and prognosis, such as CRP, ESR, autoantibodies such as anti-citrinullinated peptide antibody, and TNF levels [38–40], although these markers have not been reliable predictors of clinical responses to bDMARDs [41].

A separate post hoc analysis of clinical endpoints evaluated in the MONARCH RCT [36] demonstrated that patients with high serum IL-6 levels prior to treatment with sarilumab or adalimumab had increased baseline disease activity, joint damage, pain, and lower patient global assessment and HRQoL scores, and less likely to benefit from TNFi therapies. Furthermore, patients with high versus normal IL-6 levels reported lower responses to placebo plus methotrexate or adalimumab compared with sarilumab treatment [36].

In addition to evaluating clinical markers, quantifying the burden of RA from the patient perspective is vital to comprehensively understand the disease and its treatment [42, 43]. Findings from this present study support that baseline IL-6 levels may differentially predict treatment improvements in PRO/HRQoL.

Our findings must be examined in light of some limitations. First, the number of patients in each IL-6 tertile was modest; hence, prospective validation in larger cohorts is warranted to confirm the findings. Furthermore, while we have observed that baseline IL-6 levels predict greater improvements in PROs/HRQoL, it will be important to also assess the indirect effects of improvement of disease activity or other clinical endpoints [36] on PROs and to compare them in terms of magnitude and effect size in the different IL-6 tertiles.

Assays that measure known diagnostic biomarkers are commonly used in clinical practice (e.g., 70% of decisions made by physicians are based on results provided by biomarkers [44]). However, implementation of novel biomarkers into clinical practice proves to be a long and challenging process, which includes convincing physicians of their practicality and feasibility of use [5, 45]. Given the complexity and heterogeneous nature of RA, it is unlikely that a single cytokine level will provide sufficient discrimination to predict treatment effect. Many reliable assays are now available, predominantly multiplex formats. At present, the limitation of relying on a biomarker in RA is reflected in the disease-related complexity of immunologic networks and elucidation of the respective role and redundant effects one cytokine may have on another [5].

## Conclusion

The beneficial effects of sarilumab versus adalimumab on HRQoL were greater in patients with high IL-6 levels at baseline indicating that among adult RA patients with moderate-to-severely active RA who have had an inadequate response or intolerance to one or more DMARDs, high IL-6 levels may predict greater improvements in PROs/HRQoL than low IL-6 levels. These findings support previous analyses which have shown that across various endpoints, patients with elevated baseline IL-6 levels compared with those without responded better to sarilumab compared with methotrexate or adalimumab [36].

## Data Availability

Requests for data and materials relating to this publication can be submitted to the corresponding author.

## Abbreviations

AE: Adverse event
AM-stiffness: Morning stiffness
bDMARD: Biologic disease-modifying antirheumatic drug
BP: Bodily pain
CFB: Change from baseline
CI: Confidence interval
DMARD: Disease-modifying antirheumatic drug
FACIT: Functional Assessment of Chronic Illness Therapy
GH: General health
HRQoL: Health-related quality of life
MCID: Minimal clinically important differences
MCS: Mental component summary
MH: Mental health
IL-6: Interleukin-6
LSM: Least squares mean
OR: Odds ratio
PCS: Physical component summary
PF: Physical functioning
PRO: Patient-reported outcomes
q2w: Every 2 weeks
RA: Rheumatoid arthritis
RCT: Randomized controlled trial
RE: Role-emotional
RP: Role-physical
SC: Subcutaneous
SF: Social functioning
SF-36: Short Form 36
SD: Standard deviation
TNFi: Tumor necrosis factor α inhibitor
VT: Vitality
W: Week

## References

[1] Schett G (2018). Physiological effects of modulating the interleukin-6 axis. Rheumatology.

[2] Rose-John S (2012). IL-6 trans-signaling via the soluble IL-6 receptor: importance for the pro-inflammatory activities of IL-6. Int J Biol Sci.

[3] Tanaka T, Narazaki M, Kishimoto T (2014). IL-6 in inflammation, immunity, and disease. Cold Spring Harb Perspect Biol.

[4] Hirano T, Matsuda T, Turner M, Miyasaka N, Buchan G, Tang B, Sato K, Shimizu M, Maini R, Feldmann M (1988). Excessive production of interleukin 6/B cell stimulatory factor-2 in rheumatoid arthritis. Eur J Immunol.

[5] Burska A, Boissinot M, Ponchel F (2014). Cytokines as biomarkers in rheumatoid arthritis. Mediat Inflamm.

[6] Madhok R, Crilly A, Watson J, Capell HA (1993). Serum interleukin 6 levels in rheumatoid arthritis: correlations with clinical and laboratory indices of disease activity. Ann Rheum Dis.

[7] Abdel Meguid MH, Hamad YH, Swilam RS, Barakat MS (2013). Relation of interleukin-6 in rheumatoid arthritis patients to systemic bone loss and structural bone damage. Rheumatol Int.

[8] Song SN, Tomosugi N, Kawabata H, Ishikawa T, Nishikawa T, Yoshizaki K (2010). Down-regulation of hepcidin resulting from long-term treatment with an anti-IL-6 receptor antibody (tocilizumab) improves anemia of inflammation in multicentric Castleman disease. Blood.

[9] Feve B, Bastard JP (2009). The role of interleukins in insulin resistance and type 2 diabetes mellitus. Nat Rev Endocrinol.

[10] Danesh J, Kaptoge S, Mann AG, Sarwar N, Wood A, Angleman SB, Wensley F, Higgins JP, Lennon L, Eiriksdottir G (2008). Long-term interleukin-6 levels and subsequent risk of coronary heart disease: two new prospective studies and a systematic review. PLoS Med.

[11] Hodes GE, Ménard C, Russo SJ (2016). Integrating Interleukin-6 into depression diagnosis and treatment. Neurobiol Stress.

[12] Matcham F, Davies R, Hotopf M, Hyrich KL, Norton S, Steer S, Galloway J (2018). The relationship between depression and biologic treatment response in rheumatoid arthritis: an analysis of the British Society for Rheumatology Biologics Register. Rheumatology (Oxford).

[13] Choy EHS, Calabrese LH (2017). Neuroendocrine and neurophysiological effects of interleukin 6 in rheumatoid arthritis. Rheumatology (Oxford).

[14] Jones G, Sebba A, Gu J, Lowenstein MB, Calvo A, Gomez-Reino JJ, Siri DA, Tomsic M, Alecock E, Woodworth T (2010). Comparison of tocilizumab monotherapy versus methotrexate monotherapy in patients with moderate to severe rheumatoid arthritis: the AMBITION study. Ann Rheum Dis.

[15] Smolen JS, Beaulieu A, Rubbert-Roth A, Ramos-Remus C, Rovensky J, Alecock E, Woodworth T, Alten R (2008). Effect of interleukin-6 receptor inhibition with tocilizumab in patients with rheumatoid arthritis (OPTION study): a double-blind, placebo-controlled, randomised trial. Lancet.

[16] Burmester GR, Feist E, Kellner H, Braun J, Iking-Konert C, Rubbert-Roth A (2011). Effectiveness and safety of the interleukin 6-receptor antagonist tocilizumab after 4 and 24 weeks in patients with active rheumatoid arthritis: the first phase IIIb real-life study (TAMARA). Ann Rheum Dis.

[17] Kremer JM, Blanco R, Brzosko M, Burgos-Vargas R, Halland AM, Vernon E, Ambs P, Fleischmann R (2011). Tocilizumab inhibits structural joint damage in rheumatoid arthritis patients with inadequate responses to methotrexate: results from the double-blind treatment phase of a randomized placebo-controlled trial of tocilizumab safety and prevention of structural joint damage at one year. Arthritis Rheum.

[18] Fleischmann RM, Halland AM, Brzosko M, Burgos-Vargas R, Mela C, Vernon E, Kremer JM (2013). Tocilizumab inhibits structural joint damage and improves physical function in patients with rheumatoid arthritis and inadequate responses to methotrexate: LITHE study 2-year results. J Rheumatol.

[19] Strand V, Burmester GR, Ogale S, Devenport J, John A, Emery P (2012). Improvements in health-related quality of life after treatment with tocilizumab in patients with rheumatoid arthritis refractory to tumour necrosis factor inhibitors: results from the 24-week randomized controlled RADIATE study. Rheumatology (Oxford).

[20] Strand V, Michalska M, Birchwood C, Pei J, Tuckwell K, Finch R, Gabay C, Kavanaugh A, Jones G (2017). Impact of tocilizumab monotherapy on patient-reported outcomes in patients with rheumatoid arthritis from two randomised controlled trials. RMD Open.

[21] Strand V, Michalska M, Birchwood C, Pei J, Tuckwell K, Finch R, Kivitz AJ, Smolen JS, Burmester GR (2018). Impact of tocilizumab administered intravenously or subcutaneously on patient-reported quality-of-life outcomes in patients with rheumatoid arthritis. RMD Open.

[22] Strand V, Kosinski M, Chen CI, Joseph G, Rendas-Baum R, Graham NM, van Hoogstraten H, Bayliss M, Fan C, Huizinga T (2016). Sarilumab plus methotrexate improves patient-reported outcomes in patients with active rheumatoid arthritis and inadequate responses to methotrexate: results of a phase III trial. Arthritis Res Ther.

[23] Strand V, Reaney M, Chen CI, Proudfoot CW, Guillonneau S, Bauer D, Mangan E, Graham NM, van Hoogstraten H, Lin Y (2017). Sarilumab improves patient-reported outcomes in rheumatoid arthritis patients with inadequate response/intolerance to tumour necrosis factor inhibitors. RMD Open.

[24] Burmester GR, Lin Y, Patel R, van Adelsberg J, Mangan EK, Graham NM, van Hoogstraten H, Bauer D, Ignacio Vargas J, Lee EB (2017). Efficacy and safety of sarilumab monotherapy versus adalimumab monotherapy for the treatment of patients with active rheumatoid arthritis (MONARCH): a randomised, double-blind, parallel-group phase III trial. Ann Rheum Dis.

[25] sanofi-aventis U.S. LLC. KEVZARA® (sarilumab) prescribing information. https://www.accessdata.fda.gov/drugsatfda_docs/label/2017/761037s000lbl.pdf. Accessed 20 June 2018.

[26] European Medicines Agency. Kevzara summary of product characteristics. http://www.ema.europa.eu/docs/en_GB/document_library/EPAR_-_Product_Information/human/004254/WC500230068.pdf. Accessed 20 June 2018.

[27] Strand V, Gossec L, Proudfoot CWJ, Chen CI, Reaney M, Guillonneau S, Kimura T, van Adelsberg J, Lin Y, Mangan EK (2018). Patient-reported outcomes from a randomized phase III trial of sarilumab monotherapy versus adalimumab monotherapy in patients with rheumatoid arthritis. Arthritis Res Ther.

[28] Genovese MC, Fleischmann R, Kivitz AJ, Rell-Bakalarska M, Martincova R, Fiore S, Rohane P, Van Hoogstraten H, Garg A, Fan C (2015). Sarilumab plus methotrexate in patients with active rheumatoid arthritis and inadequate response to methotrexate: results of a phase III study. Arthritis Rheumatol.

[29] Fleischmann R, van Adelsberg J, Lin Y, Castelar-Pinheiro GD, Brzezicki J, Hrycaj P, Graham NM, van Hoogstraten H, Bauer D, Burmester GR (2017). Sarilumab and nonbiologic disease-modifying antirheumatic drugs in patients with active rheumatoid arthritis and inadequate response or intolerance to tumor necrosis factor inhibitors. Arthritis Rheumatol.

[30] Gabay C, Emery P, van Vollenhoven R, Dikranian A, Alten R, Pavelka K, Klearman M, Musselman D, Agarwal S, Green J (2013). Tocilizumab monotherapy versus adalimumab monotherapy for treatment of rheumatoid arthritis (ADACTA): a randomised, double-blind, controlled phase 4 trial. Lancet.

[31] Emery P, Rodon J, Garg A, van Hoogstraten H, Graham NM, Liu M, Parrino J, Spindler AJ, Liu N (2015). Safety and tolerability of subcutaneous sarilumab compared to intravenous tocilizumab in patients with RA. Arthritis Rheumatol.

[32] Strand V, Boers M, Idzerda L, Kirwan JR, Kvien TK, Tugwell PS, Dougados M (2011). It’s good to feel better but It's better to feel good and even better to feel good as soon as possible for as long as possible. Response criteria and the importance of change at OMERACT 10. J Rheumatol.

[33] Lubeck DP (2004). Patient-reported outcomes and their role in the assessment of rheumatoid arthritis. Pharmacoeconomics.

[34] Cella D, Yount S, Sorensen M, Chartash E, Sengupta N, Grober J (2005). Validation of the Functional Assessment of Chronic Illness Therapy Fatigue Scale relative to other instrumentation in patients with rheumatoid arthritis. J Rheumatol.

[35] Strand V, Burmester GR, Zerbini CA, Mebus CA, Zwillich SH, Gruben D, Wallenstein GV (2015). Tofacitinib with methotrexate in third-line treatment of patients with active rheumatoid arthritis: patient-reported outcomes from a phase III trial. Arthritis Care Res (Hoboken).

[36] Boyapati A, Schwartzman S, Msihid J, Choy E, Genovese MC, Burmester GR, Lam G, Kimura T, Sadeh J, Weinreich DM, et al. High Serum Interleukin-6 is Associated With Severe Progression of Rheumatoid Arthritis and Increased Treatment Response Differentiating Sarilumab from Adalimumab or Methotrexate in a Post Hoc Analysis. Arthritis Rheumatol. 2020;72(9):1456–66. 10.1002/art.41299 PMC7496495 32343882

[37] Gavrila BI, Ciofu C, Stoica V (2016). Biomarkers in rheumatoid arthritis, what is new?. J Med Life.

[38] Takeuchi T, Miyasaka N, Tatsuki Y, Yano T, Yoshinari T, Abe T, Koike T (2011). Baseline tumour necrosis factor alpha levels predict the necessity for dose escalation of infliximab therapy in patients with rheumatoid arthritis. Ann Rheum Dis.

[39] Wijbrandts CA, Dijkgraaf MG, Kraan MC, Vinkenoog M, Smeets TJ, Dinant H, Vos K, Lems WF, Wolbink GJ, Sijpkens D (2008). The clinical response to infliximab in rheumatoid arthritis is in part dependent on pretreatment tumour necrosis factor alpha expression in the synovium. Ann Rheum Dis.

[40] Daien CI, Morel J (2014). Predictive factors of response to biological disease modifying antirheumatic drugs: towards personalized medicine. Mediat Inflamm.

[41] Fabre S, Guisset C, Tatem L, Dossat N, Dupuy AM, Cohen JD, Cristol JP, Daures JP, Jorgensen C (2009). Protein biochip array technology to monitor rituximab in rheumatoid arthritis. Clin Exp Immunol.

[42] European Medicines Agency. Guideline on clinical investigation of medicinal products other than NSAIDs for treatment of rheumatoid arthritis. http://www.ema.europa.eu/docs/en_GB/document_library/Scientific_guideline/2015/06/WC500187583.pdf. Accessed 14 Nov 2016.

[43] Kirwan JR, Tugwell PS (2011). Overview of the patient perspective at OMERACT 10--conceptualizing methods for developing patient-reported outcomes. J Rheumatol.

[44] Tesch G, Amur S, Schousboe JT, Siegel JN, Lesko LJ, Bai JP (2010). Successes achieved and challenges ahead in translating biomarkers into clinical applications. AAPS J.

[45] Pirmohamed M (2010). Acceptance of biomarker-based tests for application in clinical practice: criteria and obstacles. Clin Pharmacol Ther.

